# Supplementary material for: Activity driven sleep dynamics: A conceptual modeling study
Source: PLoS One. 2026 Feb 6;21(2):e0341279. doi: 10.1371/journal.pone.0341279 (PMC12880679; doi:10.1371/journal.pone.0341279)
Supplement: S1 Appendix — (PDF) [file pone.0341279.s001.pdf]

S1 Appendix

The sleep-wake switch model we used is suggested in [1, 2] and modified in [3].

Equations (1)-(2) describe the activity of neuronal nuclei. In them, $Q(V_i)$ is a sigmoid function (7) representing the average firing rate of the population V_i at its maximum value Q_{max}, the average potential relative to rest Θ and the standard deviation $\sigma'\pi/\sqrt{3}$. The values τ_v and τ_m define the rate of change of the variables V_v and V_m, the parameters ν_{mv} and ν_{vm} define the strength of the interaction, A_v and A_m represent the influence of other populations of neurons. The parameters $\nu_{vH}H$ and $\nu_{vC}C(X,Y)$ define the strength of the influence of homeostatic and circadian processes, respectively.

The equation (3) defines the homeostatic process (variable H). Here, τ_H is the time constant, and ν_{Hm} is the strength of the effect of monoaminergic nuclei.

The circadian process C is modeled by equations (4) and (5) for variables X and Y, respectively, where $\tau_x = \tau_y$ are time constants that determine the period of the circadian oscillator, γ is a parameter that controls the oscillation shape, and τ_c and δ are introduced to match the experimental data.

The empirically selected functions C_{Xp}, C_{Yp} (expressions (8) and (9)) determine the effect of light on the circadian oscillator. In this case, the effect depends on the current phase of the circadian oscillator (parameters ν_{YY} and ν_{YX}). The sensitivity of the light component to circadian variables is determined by ϵ.

The variable P and the equation (6) describe the activity of photoreceptors — ganglion cells of the retina that affect the circadian center with light [4, 5]. The functions α_I and β define the activation rate of the accessible part of the photoreceptors $(1-P)$, and the rate of spontaneous deactivation, respectively. The dependence of the photoreceptor activity on the light intensity and the activation time constant are specified by α_0, I_0, I_1 and τ_p, respectively.

The feedback effect of neuronal nuclei on the circadian process is taken into account by the state function S, indicating whether the system is "asleep" or "awake" ($U(x)$=1 when $x > 0$ and $U(x)$=0 otherwise), as well as ν_{Xn} - the constant influence of non-photic factors. The parameter r modulates the time of effects depending on the phase of the circadian oscillator.

The equation (1) describes the influence of the circadian oscillator on the activity of the sleep center. In it, the strength of the circadian influence is given by the parameter ν_{vC}, and the shape of the nonlinear function proposed in [3] is given by the parameters c_1, c_2 and c_3.

The equations of the model (1)-(6) have the form:

$$\tau_v \frac{dV_v}{dt} = \nu_{vm} Q(V_m) - V_v + \nu_{vH} H + \nu_{vC} C(X,Y) + A_v, \tag{1}$$

$$\tau_m \frac{dV_m}{dt} = \nu_{mv} Q(V_v) - V_m + A_m, \tag{2}$$

$$\tau_H \frac{dH}{dt} = \nu_{Hm} Q(V_m), \tag{3}$$

$$\tau_x \frac{dX}{dt} = Y + \gamma \left(\frac{1}{3} X + \frac{4}{3} X^3 - \frac{256}{105} X^7 \right) + C_{Xn} + C_{Xp}, \tag{4}$$

$$\tau_y \frac{dY}{dt} = - \left(\frac{\delta}{\tau_c} \right)^2 X + C_{Yp}, \tag{5}$$

$$\tau_P \frac{P}{dt} = \alpha_I (1 - P) - \beta P. \tag{6}$$

The relations (7)-(13) included in the equations above have the form:

$$Q(V_i) = \frac{Q_{max}}{1 + e^{(\Theta - V_i)/\sigma'}},\ i = m, v, \tag{7}$$

$$C_{Xp} = \nu_{Xp} \alpha_I (1 - P)(1 - \epsilon X)(1 - \epsilon Y), \tag{8}$$

$$C_{Yp} = \alpha_I (1 - P)(1 - \epsilon X)(1 - \epsilon Y)(\nu_{YY} Y - \nu_{YX} X), \tag{9}$$

$$C_{Xn} = \nu_{Xn} \left(\frac{1}{3} - (1 - S) \right) (1 - tanh(rX)), \tag{10}$$

$$S = U(V_m - V_{th}), \tag{11}$$

$$C(X,Y) = 0.1 \frac{(X+1)}{2} + \left(\frac{c_1 X - c_2 Y + c_3}{X + 2} \right)^2, \tag{12}$$

$$\alpha_I = \alpha_0 S \frac{I(t)}{I(t) + I_1} \sqrt{\frac{I(t)}{I_0}}. \tag{13}$$

The daily illumination profile $I(t)$ corresponds to a signal of the "meander" type, where the model control parameters I_{amb} and I_{ext} set the night and day intensity, respectively.

The meaning of the model parameters is presented below: Q_{max}= 100 Hz; Θ = 10 mV; σ' = 3 mV; $\tau_v = \tau_m$ = 50/3600 h; ν_{vm} = -2.1/3600 mV h; ν_{mv} = -1.8/3600 mV h; A_v = -10.3 mV; A_m = 1.3 mV; V_{th} = -2 mV ; ν_{vH} = 1 mV; ν_{vC} = -0.5 mV; τ_H = 59 h; ν_{Hm} = 4.57/3600 h; $\tau_x = \tau_y$ = 24/(2π) h; γ = 0.13; τ_c = 24.2 h; δ = 24.2/0.99729 h; c_1= 0.838; c_2 = 0.676; c_3 = 1.136; ν_{Xn} = 0.032; r = 10 h; ν_{Xp} = 2220; ν_{YY} = 739.8 h; ν_{YX} = 1221 h; ϵ = 0.4; β = 0.007/60 Hz; α_0 = 0.1/60 Hz; I_0 = 9500 lx; I_1 = 100 lx; I_{amb} = 0 lx; I_{ext} = 100 lx; τ_p= 1/3600 h.

References

[1] Phillips A, Robinson PA. A quantitative model of sleep-wake dynamics based on the physiology of the brainstem ascending arousal system. Journal of Biological Rhythms. 2007;22(2):167–179.

[2] Hilaire MAS, Klerman EB, Khalsa SBS, Wright JrKP, Czeisler CA, Kronauer RE. Addition of a non-photic component to a light-based mathematical model of the human circadian pacemaker. Journal of theoretical biology. 2007;247(4):583–599.

[3] Postnova S, Lockley SW, Robinson PA. Sleep propensity under forced desynchrony in a model of arousal state dynamics. Journal of biological rhythms. 2016;31(5):498–508.

[4] Berson DM. Strange vision: ganglion cells as circadian photoreceptors. TRENDS in Neurosciences. 2003;26(6):314–320.

[5] Wong KY, Dunn FA, Graham DM, Berson DM. Synaptic influences on rat ganglion-cell photoreceptors. The Journal of physiology. 2007;582(1):279–296.
