# Supplementary material for: Activity driven sleep dynamics: A conceptual modeling study
Source: PLoS One. 2026 Feb 6;21(2):e0341279. doi: 10.1371/journal.pone.0341279 (PMC12880679; doi:10.1371/journal.pone.0341279)
Supplement: Attachment — Submitted filename: rebuttal_letter_R1.pdf [file pone.0341279.s002.pdf]

Rebuttal letter

We are grateful to the reviewers for their positive assessment of our study and helpful suggestions for improvement. Below are our responses to each of the reviewers' comments.
All changes made to the manuscript are highlighted in blue.

Reviewer #1:
Line 6: “extracellular fluid”
Our response:
It was rephrased: “Thus, it has been shown that during deep sleep, the volume of extracellular space increases significantly.”

Line 55: “Kruger says...” What does he say? Rewrite sentence
Our response:
It was rephrased as follows: “In the paper \cite{krueger2023tripping} the author expresses the opinion that "sleep is a property and is initiated by small groups of closely interconnected neurons."

Line 61: On what time scale?
Our response:
It was rephrased: “From a temporal perspective, the main challenge for a two-process model is that it does not assume "extra" short episodes of sleep. “

Line 78: Remove “be”
Our response:
It has been done

Line 303: how efficient the proposed structures (are)
Our response:
It was rephrased:
“Since we propose the model structure from general considerations, although taking into account physiology, a numerical experiment is necessary to evaluate the effectiveness of the proposed structures.”

Line 483 “substantial” should be “substantially”
Our response:
It was corrected.

Additionally, for Figure 2,4,6,7 the x axis should be clearly labeled (what are t and h?)
Our response:
The x-axis in Figures 2,4,6,7 has been clarified (also in one of the graphs in Figure 5).
The designation "t, h" has been changed to "Time, hours".

Reviewer #2:

Major
1. The model is currently presented as a conceptual proof-of-principle. To enhance its impact, the authors should show its potential applications and the pathways for its future validation and quantitative development.

Our response:
Further validation of the model is desirable at various levels. Specifically, at the level of an individual NGVU, it is necessary to clarify the nature of state changes (e.g., the dynamics of gliotransmitter release) during overconsumption of metabolic reserves, such as astrocytic glycogen.
At the system level, further validation of the model would highly require objective data on brain activity patterns in simulated situations that activate and overload as few neural structures as possible. This direction is the most promising, as it also offers potential for practical applications. Just as the two-process model was adapted for purely practical tasks on a daily time scale,
our model may also be useful for studying the more rapid processes of drowsiness episodes during prolonged periods of concentration (long night drives, dispatchers working at a control panel, etc.).

Another important potential application is the use of the model as one of the tools in a comprehensive study of the possibility of non-simultaneous (local, sequential) activation of brain cleaning. It has already been shown that the phenomenon of local sleep is associated with local accelerated recovery after cognitive load.
It is conceivable that during prolonged sleep deprivation, such local events could serve to protect the brain and, to a certain extent, replace global sleep. Of course, experimental work is crucial here, but a suitable modeling paradigm is also important.

Regarding the quantitative development of the model, there are at least two approaches focused on different goals. Specifically, at the level of cellular structures, there are known studies on quantitative modeling of processes in NGVU. This makes it possible to quantify molecular transport in the parenchyma.

However, the authors believe that accurate quantitative timing of the local portion of the model is a more attractive and achievable goal, similar to what was done for the two-process model. The main challenge here is to accurately estimate the characteristic timing of processes such as various signaling pathways, for example, from parenchymal astrocytes to centers that sense sleep propensity.

We have included everything described above in the Discussion text.

2. The discussion of limitations is good but could be expanded to include, for example, the model's current lack of sleep stage differentiation (NREM/REM) and more concrete hypotheses for its application to brain clearance mechanisms.

Our response:
We are grateful to the reviewer for this very pertinent comment. We indeed failed to address this issue in the previous version of the manuscript.
In the revised manuscript, we have supplemented the Introduction as follows:

Note that there is a general consensus that activation of harmful metabolite clearance during sleep is due to an increase in the proportion of extracellular space, which facilitates the transport of any molecules within the parenchyma. Since this increase occurs at low neural activity, all our considerations assume a state of deep sleep, NREM sleep, when SWA is recorded in the electroencephalogram.

We have also expanded the Discussion as follows:

As mentioned in the Introduction, one limitation of the model is that it does not distinguish between REM and NREM sleep states. Note that this is not possible in the original model of the two processes, which we supplement with "low-level" components.
Specifically, since REM sleep is characterized by high levels of neural activity, it contributes little (if anything) to accelerating the clearance of harmful metabolites and does not fit with our current working hypothesis. In our view, a different organizational level of model construction is required to adequately incorporate REM-NREM transitions. This could presumably be achieved by identifying typical spatial patterns of activity during REM sleep and associating them with various "medium-sized" sleep modules. Currently, the authors are unaware of experimental data that would allow for the valid construction of such a model.

Minor
1. The manuscript requires minor language edits. For example,
Line 79, "in the spirit of" what?
Our response:
It was rephrased: "Next, we introduce a psycho-sensory drive signal into the model following the ideas expressed in the work ~\cite{george2018psycho}. "

Line 210, missing "is" after "D";
Line 257, "Level" =>" level";
Line 532, "Ca2+" =>" Ca^2+".
Our response:
It has been done.
